# The Current Status of Molecular Biomarkers for Inflammatory Bowel Disease

**DOI:** 10.3390/biomedicines10071492

**Published:** 2022-06-24

**Authors:** Zahra Alghoul, Chunhua Yang, Didier Merlin

**Affiliations:** 1 Digestive Diseases Research Group, Institute for Biomedical Sciences, Georgia State University, Atlanta, GA 30303, USA; zalghoul@gsu.edu (Z.A.); dmerlin@gsu.edu (D.M.); 2 Department of Chemistry, College of Arts & Sciences, Georgia State University, Atlanta, GA 30303, USA; 3 Atlanta Veterans Affairs Medical Center, Decatur, GA 30033, USA

**Keywords:** proteomics, epigenetics, endoscopy, imaging, laboratory testing

## Abstract

Diagnosis and prognosis of inflammatory bowel disease (IBD)—a chronic inflammation that affects the gastrointestinal tract of patients—are challenging, as most clinical symptoms are not specific to IBD, and are often seen in other inflammatory diseases, such as intestinal infections, drug-induced colitis, and monogenic diseases. To date, there is no gold-standard test for monitoring IBD. Endoscopy and imaging are essential diagnostic tools that provide information about the disease’s state, location, and severity. However, the invasive nature and high cost of endoscopy make it unsuitable for frequent monitoring of disease activity in IBD patients, and even when it is possible to replace endoscopy with imaging, high cost remains a concern. Laboratory testing of blood or feces has the advantage of being non-invasive, rapid, cost-effective, and standardizable. Although the specificity and accuracy of laboratory testing alone need to be improved, it is increasingly used to monitor disease activity or to diagnose suspected IBD cases in combination with endoscopy and/or imaging. The literature survey indicates a dearth of summarization of biomarkers for IBD testing. This review introduces currently available non-invasive biomarkers of clinical importance in laboratory testing for IBD, and discusses the trends and challenges in the IBD biomarker studies.

## 1. Introduction

Inflammatory bowel disease (IBD) is a set of chronic and idiopathic inflammatory conditions that affect more than 3.5 million patients worldwide. The two major forms of IBD are Crohn’s disease (CD), in which inflammation affects any segment of the gastrointestinal (GI) tract [1], and ulcerative colitis (UC), in which inflammation affects the inner lining of the colon or rectum [2]. Patients with IBD are up to six times more likely to develop colorectal cancer than the general population [3,4]. In addition to the molecular alterations (such as chromosomal instability, microsatellite instability, and hypermethylation) that contribute to sporadic colorectal cancer, IBD-related colorectal cancer is linked to inflammation that induces the transcription of mutated cancer genes [5]. Loss-of-function mutations in tumor-suppressor protein p53 occur in both sporadic and IBD-related colorectal cancer, but they occur earlier in the non-dysplastic mucosa of IBD-related colorectal cancer than in sporadic colorectal cancer [4,5]. Another mutation observed in both types of cancer is the nonfunctional adenomatous polyposis coli (APC) gatekeeper gene. Unlike the p53 mutation, APC mutation occurs just prior to carcinoma in IBD-related colorectal cancer, but at a much earlier stage in sporadic colorectal cancer [4]. Other gene mutations linked to IBD-related colorectal cancer include p27, k-Ras (12p12) oncogene, human mismatch repair genes (e.g., hMLH1, hMSH2), and p16 [4].

CD and UC are both characterized by mucosal inflammation, with occasional flares and remittance. Inflammation in CD can affect any segment of the GI tract, and spreads in a non-continuous pattern [1,6]. CD commonly involves the formation of strictures, abscesses, and fistulas [6]. Its histological features include thickened submucosa, fissuring ulceration, transmural inflammation, and non-caseating granulomas [6]. Inflammation in UC affects the inner lining of the colon or rectum, and spreads in a continuous pattern [2,6]. It shows superficial inflammatory changes in the mucosa and submucosa, and involves the formation of cryptitis and crypt abscesses [6]. The clinical symptoms of IBD include abdominal pain, diarrhea, rectal bleeding, weight loss, nausea, intestinal pain and, in some cases, fever [7,8]. As these symptoms are not specific to IBD, the clinical diagnostic process must consist of using a combination of endoscopic, radiological, clinical, histological, and laboratory tests [9]; a single technique is often insufficient for the diagnosis.

Endoscopy and imaging are essential techniques for the diagnosis, management, and treatment of IBD. They are used in the initial evaluation of patients with suspected IBD, as well as in making a differential diagnosis of UC versus CD in confirmed IBD cases [10]. The strength of endoscopy as a diagnostic tool lies primarily in its ability to visually observe different bowel segments, allowing clinicians to assess disease severity and monitor disease activity over time. Ileocolonoscopy has traditionally been the most used form of endoscopy in IBD. The initial evaluation of patients presenting with clinical symptoms suggestive of IBD should be carried out with ileocolonoscopy, as recommended by the American Society for Gastrointestinal Endoscopy (ASGE) Standards of Practice Committee [11]. In addition to providing a visual of the colon and the terminal ileum, ileocolonoscopy can be used to obtain biopsy specimens for further analysis. The ASGE suggests obtaining at least two biopsy specimens from five sites throughout the bowel during the initial evaluation [12]. However, the invasiveness and high cost of ileocolonoscopy are major drawbacks that have limited its frequent use for monitoring disease activity.

New, less-invasive endoscopic techniques that can more accurately diagnose IBD, while also providing a differential diagnosis of CD and UC, have emerged in the past few years. These include video capsule endoscopy (VCE), confocal laser endomicroscopy (CLE), and single- or double-balloon-assisted enteroscopy (SBE and DBE, respectively). VCE provides imaging of the whole bowel via ingestion of a wireless capsule endoscope [13]. This technique is particularly useful for inspecting areas in the GI tract that cannot be visualized by colonoscopy [14]. Although the risk of capsule retention is low, it remains the primary concern in patients with suspected or known IBD [15]. VCE is less invasive and more cost-effective than ileocolonoscopy, but it cannot be used in performing biopsies. In CLE, a confocal laser microscope is used in vivo to obtain living tissue images during colonoscopy [16]. CLE has the advantage of offering a faster diagnosis than a traditional colonoscopy. Enteroscopy in both of its forms (SBE and DBE) allows access to small bowel areas that standard endoscopy cannot reach. Additionally, enteroscopy can be used in performing histological analysis. However, due to its technical complexity and time-consuming preparation, enteroscopy is not recommended for the initial evaluation of suspected IBD cases [17].

In confirmed IBD cases, clinical symptoms alone are insufficient for clinicians to determine the extent of mucosal inflammation, or to make a differential diagnosis between UC and CD. There has been a growing interest in the use of cross-sectional imaging modalities such as magnetic resonance enterography (MRE), ultrasonography (US), and computed tomography (CT) as tools to supplement endoscopy in the diagnosis and monitoring of IBD [18]. These techniques are instrumental in detecting mural and extramural complications and assessing laminal inflammation in areas affected by CD in the small bowel that are beyond the reach of colonoscopy [19]. Due to their ability to diagnose CD with high accuracy, cross-sectional imaging modalities are used to make differential diagnoses in suspected cases of UC [20]. This aspect is critical because these diseases differ in their prognosis and required treatments.

Although imaging techniques offer highly accurate IBD diagnosis, they require experienced personnel, sophisticated instruments, and high costs, hampering their routine application. Laboratory testing’s advantage lies in the fact that these tests can be standardized, rapid, and cost-effective, but they can also be applied to the already established patient sample libraries to process independent investigations. An increasing number of laboratory tests, combined with endoscopy or imaging, are used to monitor disease activity or diagnose suspected IBD cases. As good laboratory test results rely on the proper use of molecular biomarkers from the patients’ tissue, blood (serum), or fecal samples, this review summarizes currently available biomarkers of clinical importance in laboratory testing of IBD, discusses the possible involved genetic and epigenetic factors, and envisions the trends and challenges of biomarker discovery in IBD.

## 2. Non-Invasive Molecular Biomarkers of IBD: Serum Proteins, Serological Antibodies, and Fecal Proteins

**Figure 1:**
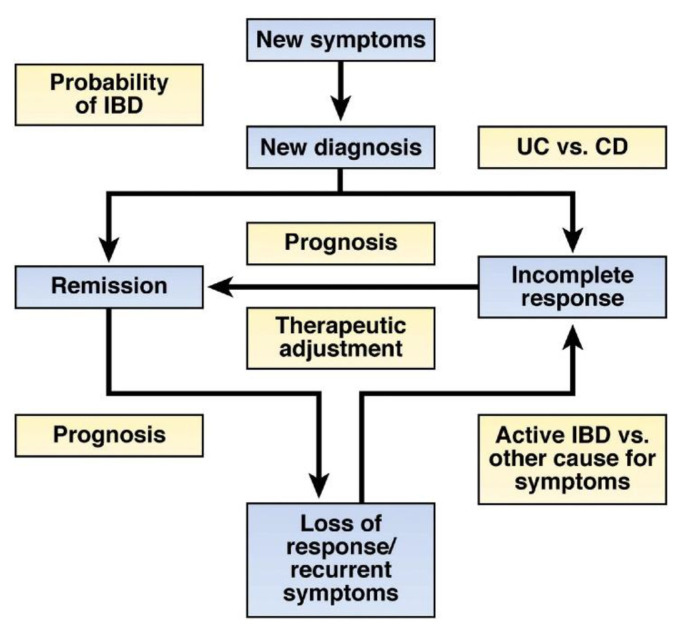
The potential role of biomarker assays in the care of patients with suspected or established IBD: Biomarkers may be used in all phases of the care. For patients with suspected IBD, biomarkers can be used to select which patients are unlikely to have IBD and could forgo further testing. Once patients are diagnosed, biomarkers can determine which patients have CD or UC and predict the disease course. Biomarkers can be used to determine which patients are most likely to respond to therapies, determine prognosis, and identify those who require more aggressive therapies. In patients with recurrent symptoms, biomarkers can differentiate patients with active inflammation from those likely to have symptoms from other causes. Adapted from James D. Lewis’s review [23]; *Gastroenterology*, Volume 140 Issue 6, Pages 1817–1826.e2; https://doi.org/10.1053/j.gastro.2010.11.058.

Biomarkers play critical roles in the early detection and monitoring of disease progression and therapeutic responses (Figure 1). Disease activity can be monitored with laboratory tests that measure circulating biomarkers in the blood (serum or plasma), tissue, or feces. A biomarker is defined as “a characteristic that is objectively measured and evaluated as an indicator of normal biological processes, pathogenic processes, or pharmacological responses to a therapeutic intervention” [21]. Identifying a biomarker or several biomarkers of a given condition’s pathologies might help to diagnose, prognose, and assess therapeutic responses. For a biomarker to be effective, it should possess several attributes, such as being non-invasive, inexpensive, convenient for sampling, reproducible, and disease-specific (i.e., accurate and precise). An ideal biomarker also needs to have a rapid test-to-result turnaround time, be standardizable to provide comparable test results across different assays, be widely available and stable for storage, have a wide dynamic range, use defined thresholds to determine the absence/presence or extent of inflammation, and be responsive to changes in the state of inflammation [22].

Several molecular biomarkers have been established as reliable measures for disease activity in IBD [22,24]. They are minimally invasive and relatively inexpensive compared to colonoscopy and imaging techniques. They can also assist in identifying patients who require diagnosis with endoscopy and biopsies. However, many of these biomarkers have limitations in terms of their specificity, sensitivity, responsiveness, and/or other desirable attributes of IBD biomarkers [22]. There are currently three major types of molecular biomarkers available for IBD: serum biomarkers, serological antibodies, and fecal biomarkers.

### 2.1. Serum Biomarkers

Several inflammatory serum biomarkers have become part of routine laboratory testing for the diagnosis of IBD. Although they are not specific to IBD, these serum biomarkers are commonly used for initial diagnosis due to their ease of use, low cost, and well-established protocols. The most common of these tests are those for C-reactive protein (CRP) and the erythrocyte sedimentation rate (ESR).

CRP is a pentameric protein that is produced in the liver by hepatocytes. It is found in serum at <1 mg/L under physiological conditions. Its concentration increases during an acute-phase response, as pro-inflammatory cytokines such as IL-6, tumor necrosis factor α (TNF-α), and IL-1β stimulate its production in the hepatocytes [25,26,27]. CRP has a relatively short half-life (about 19 h) [28], making it a better indicator of inflammation than most acute-phase proteins. Elevated CRP levels are observed in most active CD cases, whereas the CRP levels of UC patients show little-to-no increase in the case of active disease [27,29]. This may reflect the production of CRP by mesenteric adipocytes in patients with CD [30]. Although CRP is widely used as a biomarker for IBD, it lacks specificity; elevated CRP levels are also observed in autoimmune disorders, infections, and malignancies [25].

ESR is a measure of how quickly erythrocytes sediment through plasma in a column, with a higher rate taken as indicating more inflammation. ESR values are affected by physiological factors such as pregnancy, age, and gender, as well as changes in hematocrit levels in patients with anemia and polycythemia [31]. Medications that cause changes in the size of erythrocytes can also affect ESR values [32]. Changes in ESR values are not specific to IBD, and can be due to any inflammatory stimulus. Unlike CRP, ESR values are altered in both UC and CD, and we cannot distinguish them. ESR values peak more slowly than CRP, and take longer to return to normal after the end of an inflammatory flare [28].

CRP and ESR have been studied long enough to become established in IBD diagnosis. While both tests lack the specificity and accuracy to be considered a gold-standard diagnosis, CRP has some advantages over ESR. For example, the CRP concentration changes faster than the ESR value upon a change in disease activity, CRP has a broader range of abnormal values than ESR, and (unlike ESR) CRP does not show age-related variation [33].

Leucine-rich alpha-2 glycoprotein (LRG) is a 50 kD protein that is secreted by hepatocytes, neutrophils, macrophages, and intestinal epithelial cells [34,35,36]. It has recently emerged as a novel serological biomarker for IBD and rheumatoid arthritis. Studies have found that levels of LRG are elevated in patients with active UC, and decrease with a decline in disease activity [37,38]. Notably, elevated levels of LRG correlate better than CRP with clinical and endoscopic scores in patients with active UC and CD [38,39,40]. LRG has been also found to predict mucosal healing in both UC and CD patients with normal CRP levels [41].

### 2.2. Serological Antibodies

Serological testing is a well-established diagnostic tool for a variety of immune diseases. Its use in IBD has been mainly focused on patients with a confirmed diagnosis; little work has been done on its potential as a primary diagnostic tool in patients with suspected IBD. Perinuclear anti-neutrophil cytoplasmic antibodies (p-ANCAs) and anti-*Saccharomyces cerevisiae* antibodies (ASCAs) are the two primary antibodies currently examined in IBD studies. ANCAs are a group of antibodies produced against antigens in the cytoplasm of neutrophils. ASCAs are produced against mannan and other yeast cell wall components. Both have been reported to provide clinically useful positive or negative predictive values: p-ANCA+/ASCA− is reported in patients with UC, while p-ANCA−/ASCA+ is seen in patients with CD. Although each of these biomarker antibodies can be used to discriminate UC from CD, they both have low accuracy and sensitivity [42]. Positive results for either antibody are not unique to IBD, and may be related to several other GI and inflammatory conditions, such as celiac disease, Behcet’s disease, cystic fibrosis, and rheumatoid arthritis [42,43].

### 2.3. Fecal Biomarkers

Fecal biomarkers are the proteins that are explicitly found in stool samples of patients with IBD. The fecal biomarkers for IBD reported to date are mainly fecal leukocyte proteins. These include calprotectin, calgranulin C, lactoferrin, and lipocalin-2. They have several advantages over blood biomarkers, including the ease of sample accessibility, high biomarker concentration due to the direct contact of the fecal sample with the site of inflammation, and higher specificity for IBD because they reflect GI inflammation (unlike serum biomarkers, which are increased by various types of inflammation) [44].

Calprotectin is the most widely used fecal biomarker for IBD. It is a calcium- and zinc-binding protein that is abundant in neutrophils, eosinophils, and macrophages. Changes in its concentration are observed in various secretory and excretory products in the body upon activation of granulocytes and mononuclear phagocytes [45]. Elevated fecal calprotectin levels are expected in patients with active IBD, due to the presence of a high number of neutrophils in the GI tract, which is characteristic of the disease [28]. Calprotectin is resistant to degradation, and is stable for 7 days in fecal samples stored at room temperature [46]. Changes in fecal calprotectin levels are not exclusive to IBD; alterations are also observed in various colon and intestine diseases [47].

Calgranulin C (S100A12) belongs to the S100 family of low-molecular-weight calcium-binding proteins, which activate the NF-κB pathway and increase cytokine release during pro-inflammatory processes [31]. The serum concentration of calgranulin C is high in IBD [48], but the fecal concentration is higher, making the fecal assay more sensitive to IBD. Elevated levels of calgranulin C have been reported in other inflammatory conditions, such as arthritis [49].

Lactoferrin is another biomarker whose levels are significantly elevated in active IBD. It is an iron-binding glycoprotein that is found specifically in neutrophils; in this respect, it contrasts with calprotectin, which is found in several types of cells. Lactoferrin has high specificity and sensitivity for diagnosing active IBD [50].

Lipocalin-2 (LCN-2), also known as neutrophil gelatinase-associated lipocalin (NGAL) or siderocalin (Scn), is a bacteriostatic protein stored in neutrophil granules [51,52]. LCN-2 is involved in innate immunity by secluding iron from pathogenic bacteria, limiting their invasion. It is a highly stable protein whose elevated expression by gut epithelial cells has been demonstrated in colonic biopsies from inflamed areas of patients with IBD. Serum LCN-2 has been proven to be an active biomarker in UC patients, and it is widely used as a fecal biomarker of acute inflammation in the animal model of UC, indicating that it can potentially be used as a fecal biomarker of human UC. Upregulation of LCN-2 is believed to be induced by IL-22 and IL-17A [53].

### 2.4. Diagnostic/Prognostic Accuracy

The major concern about diagnosis and prognosis of IBD that solely rely on singular molecular biomarkers is their detection accuracy. A study showed that the biomarkers’ correlation coefficients with endoscopy could vary from 0.48 to 0.83 (for calprotectin) and from 0.19 to 0.87 (for lactoferrin) in IBD patients [23] (Table 1). IBD detection methods that combine endoscopy with histopathology biomarkers can be highly accurate, such as in the context of oncostatin M (OSM) or oncostatin M receptor (OSMR), which are found to be highly overexpressed in the inflamed intestinal tissue of active IBD patients, with a *p*-value < 0.001 for OSM (*n* = 42) and a *p*-value < 0.05 for OSMR (*n* = 86) at a false discovery rate (FDR) of 1% [54].

**Figure 2:**
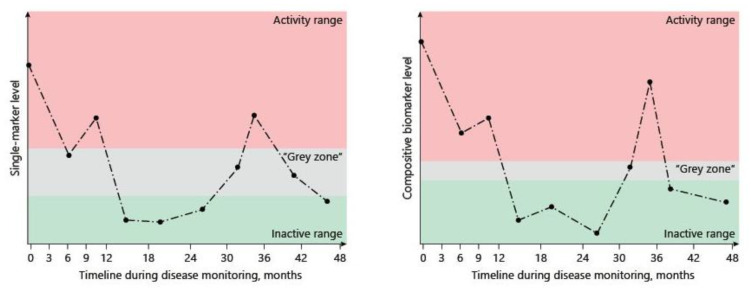
Improvements are provided by composite biomarkers. Careful selection of markers and their integration can optimize the diagnostic accuracy of single biomarkers of disease activity and drastically reduce the blind spot resulting from the “grey zone”. Adapted from Dragoni G. et al.’s review [55]; *Digestive Diseases*, https://doi.org/10.1159/000511641.

To date, C-reactive protein and fecal calprotectin are considered reliable markers of disease activity, with demonstrated utility in IBD management [55]. However, single-biomarker-based detections often present a larger ambiguous “grey zone” than detections made using composite biomarkers (Figure 2). Composite biomarkers are defined as “a combination of ≥2 biomarkers”, and are selected using an optimized algorithm to render a single interpretive output. The combination of different biomarkers has shown higher accuracy, and is expected to reduce the “grey zone” of each biomarker and replace single-marker approaches in the future of research and clinical practice [55] (Figure 2).

## 3. Trends in IBD Biomarker Discovery

### 3.1. Proteomics

Proteomics, the study of the set of gene-encoded proteins known as the proteome, uses a range of techniques for separating, identifying, and structurally characterizing proteins. Proteomics goes beyond the study of proteins in a given cell, including their isoforms, post-translational modifications, and protein–protein interactions [56]. Depending on the analysis method, proteomic approaches can be bottom-up or top-down. In bottom-up proteomics, proteolytic digestion breaks the extracted proteins into peptides, which are then analyzed by mass spectrometry (MS). In top-down proteomics, intact proteins are analyzed. The samples used in IBD-related studies are usually obtained from blood (serum or plasma) or colonic biopsies. Liquid chromatography coupled with electrospray tandem mass spectrometry (LC–ESI-MS/MS) is the most widely used proteomic technique in IBD research. Other commonly used techniques include two-dimensional gel electrophoresis coupled with matrix-assisted laser desorption/ionization (MALDI)-MS screening and immunofluorescence microscopy.

Due to the strong connections between protein expression and disease activity, the application of proteomics in biomarker discovery is a promising emerging field. Advances in LC–MS instrumentation, such as the combination of ultrahigh-performance liquid chromatography (UPLC) with nano-electrospray ionization and high-resolution mass spectrometry (HRMS), have revealed the potential of MS-based proteomics to compete with or even replace traditional immunoassay techniques. It is hoped that proteomics may help to develop personalized and precision medicine [57]. Instead of focusing on finding a single biomarker, current proteomic biomarker research aims to identify protein biomarker panels representing an individual’s disease state. In this context, three approaches have emerged over the past few years: (1) Proteotyping—a multiprotein approach used to determine an individual’s unique proteome [58]. (2) Proteogenomics—a multi-omics approach in which genomic and proteomic analyses are performed on the same sample; data obtained from this pairing contain information that would not be obtained using either technique alone [59,60]. (3) Proteoforms—protein variants that result from post-translational modifications of proteins, genetic mutations, or truncations. MS immunoassays are often used to map a specific protein’s proteoforms to distinguish between normal and clinical fluctuations [61,62].

To date, the use of proteomics in IBD has focused on three areas: identifying novel protein biomarkers for diagnosis, understanding the pathological mechanisms underlying disease activity, and monitoring the response to treatment. Berndt et al. pioneered the use of proteomics in IBD by performing proteomic analysis of normal and inflamed intestinal mucosa using multi-epitope ligand cartography immunofluorescence microscopy. The authors found that different T-cell populations in the mucosa expressed distinct proteins in each form of IBD [63]. An experimental approach based on combining discovery proteomics with targeted verification experiments successfully assessed transmural intestinal complications in CD, with 70% sensitivity and 72.5% specificity. This approach, which used label-free LC–MS/MS, identified a serological biomarker panel that could discriminate complicated CD from uncomplicated CD, rheumatoid arthritis, UC, and healthy controls [64]. Another study that used LC–MS identified a panel of four proteins that could distinguish active pediatric IBD from non-IBD with high sensitivity and specificity.

Additionally, the study found that two of the identified proteins were elevated in IBD stool samples, demonstrating that fecal samples can be used for measuring these biomarkers [65]. Several studies attempted to identify differentially expressed proteins in patients with UC and CD through proteomic profiling of serum or colonic biopsies. Proteomic profiling of colon biopsies using MALDI-MS identified distinct protein peaks for UC and CD specimens, indicating that it could be possible to differentially diagnose these IBD forms using protein profiles [66,67,68]. In a study that compared the proteomic spectra of submucosal samples from inflamed UC versus CD and uninflamed UC versus CD, two distinct peaks were identified in the first case, and three in the second [66]. Another study identified a set of 25 proteins as differentiators for UC and CD in colonic mucosal tissue samples obtained from 62 patients with confirmed UC/CD [67]. Screening of mucosal biopsies obtained from children with suspected IBD identified two distinct biomarker panels: one consisted of 5 proteins that were reported to discriminate IBD from control patients, while the other consisted of 12 proteins reported to allow the differential diagnosis of CD and UC patients [68]. Protein profiling of 120 serum samples from patients with CD or UC and inflammatory and healthy controls was performed using surface-enhanced laser desorption/ionization–time-of-flight mass spectrometry (SELDI-TOF-MS). This work identified four diagnostic protein biomarkers for IBD, one of which could reportedly discriminate UC from CD with accuracies similar to or higher than those of the ANCA and ASCA serological tests [69]. Proteomic profiling of stricturing CD, non-stricturing CD, and UC patients identified a smaller set of peptides for differentiating stricture versus non-stricture CD in IBD [70].

In addition to diagnostic biomarkers, several studies have used proteomics to identify biomarkers that could be used to assess treatment responses in IBD. One study monitored the treatment response to infliximab in IBD patients by measuring the levels of circulating chemokines and monocyte activation using LC–nano-ESI-MS/MS. The study found that 2 weeks from the start of treatment, decreases were evident in the levels of macrophage-derived CD14 and CD86, as well as the chemokine, CCL2 potentially providing a mechanistic explanation for why not all patients respond to this treatment [71]. Another study investigated the treatment response to infliximab and prednisone in children with IBD. The study identified 18 proteins and 3 miRNAs that were responsive to both drugs; some were downregulated with inflammation, while others were upregulated as the inflammation was resolved [72].

### 3.2. Genetics

Pathological studies of IBD and its two subtypes suggest a genetic risk factor behind the immune response to the intestinal microbiota. Genome-wide association studies (GWASs) have identified approximately 240 gene loci associated with susceptibility to IBD [73]. Several studies have used genetic profiling of blood samples to identify gene panels that may help to differentiate IBD from healthy controls [74], active from inactive CD [75], and CD from UC [76,77,78]. Distinct gene panels were also identified in peripheral blood samples from pediatric IBD patients in clinical remission compared to healthy controls [79]. Other studies performed gene expression analysis on mucosal biopsies from IBD patients, and identified distinct gene panels for IBD versus healthy controls [80] and UC versus healthy controls [81]. The use of genetics to identify loci associated with IBD can potentially define causal disease mechanisms, which could, in turn, advance the biomarker discovery process [82].

### 3.3. Epigenetics

Epigenetics, which describes changes in gene function caused by gene–environment interactions rather than changes in the DNA sequence, is gaining research interest among scientists seeking to study the pathogenesis and diagnosis of IBD [83,84]. DNA methylation and RNA interference are the two most heavily researched areas in IBD epigenetic studies.

DNA methylation refers to adding a methyl group to cytosine residues in the CpG dinucleotide sequence [85]. Early studies of DNA methylation changes in the mucosa of IBD patients focused primarily on their use as predictors of malignancy [86]. Recent studies have shown that the DNA methylation of specific genes plays a role in the pathogenesis of IBD, suggesting that they could be useful as biomarkers [87,88]. A genome-wide methylation profiling conducted on rectal biopsies identified panels of genes (e.g., *THRAP2*, *FANCC*, *GBGT1*, *DOK2*, *TNFSF4*, *TNFSF12*, and *FUT7*) that showed evidence of differential methylation in CD and UC specimens in comparison to those from healthy controls [88]. Another study identified seven differentially methylated CpG sites in the diseased intestinal tissue of IBD patients compared to normal intestinal tissue from the same patients [89]. Genome-wide changes in DNA methylation have also been analyzed using the peripheral blood of patients with IBD. Analysis of the DNA methylation changes using peripheral blood from CD patients identified 50 genes that showed significant differential methylation compared to that in healthy controls [87]. Site-specific DNA methylation changes in genes associated with IBD pathways have also been identified, with the results showing a 45% overlap of the differentially methylated positions in UC and CD [90].

MicroRNAs (miRNAs) are non-coding, single-stranded RNA species that consist of 18–25 nucleotides. Disruptions in their expression profiles and function are observed in human diseases such as cancer and neurological, cardiovascular, and autoimmune diseases [91]. The potential of miRNAs as diagnostic biomarkers and treatment options in IBD has garnered growing interest in the past few years. Colonic tissue and circulating miRNAs (e.g., serum, feces) are the two types of samples used in most of these studies.

Several studies have successfully identified distinct miRNA profiles reflecting the up- or downregulation of one or more miRNAs in colonic biopsy specimens of IBD patients [92] (Table 2). One of the pioneering studies in this area identified the differential expression of 11 miRNAs in the mucosal tissue samples of patients with active UC [93]. Other studies that examined the colonic mucosa of patients with active UC reported upregulation of one or more miRNAs (such as miR-21 [94], miR-150 [95], and miR-155 [94]) and downregulation of others (such as miR-143 and miR-145 [96]), in comparison to healthy controls. Similarly, some studies compared the colonic mucosa of patients with active CD to healthy controls, and reported upregulation of miR-196 [97] and downregulation of miR-7 [98]. Other studies assayed the expression of hundreds of miRNAs, and identified panels differentially expressed in the colonic tissues of patients with UC and CD versus controls [99,100,101].

Distinct profiles of circulating miRNAs have also been identified in blood samples of IBD patients. Several studies identified many upregulated or downregulated miRNAs in peripheral blood samples from patients with IBD. Samples were obtained from patients with UC or CD versus healthy controls [101,102,103,104] and pediatric CD versus healthy controls [105]. Distinct panels of miRNAs have also been identified in fecal samples of IBD patients [106,107,108]. More investigation into the specificity of miRNAs for IBD is required before they can be used as diagnostic tools, as some miRNAs are known to be associated with other conditions. For example, miR-21 is significantly high in the blood of UC patients [103], but is also upregulated in patients with colorectal cancer [109]. One study examined the differential expression of miRNAs between UC and CD in saliva, in addition to blood and colon tissue samples [110]. The study identified several miRNAs (i.e., miR-21, miR-31, miR-142-3p, miR-142-5p) whose expression levels in all three types of samples were significantly altered between IBD and non-IBD patients.

## 4. Challenges and Future Directions

### 4.1. Proteomic Biomarker Discovery

The typical protein biomarker discovery and validation process consists of six phases: discovery, qualification, verification, assay optimization, clinical evaluation/validation, and commercialization [111]. During the discovery phase, researchers identify a list of 20 to several hundred proteins that are differentially expressed between healthy and disease-confirmed samples. This identification process is based on an unbiased, semi-quantitative assessment of peptide abundances in both samples. In the next phase, qualification, this unbiased approach is replaced with a targeted analysis to confirm the differential expression of the candidate proteins identified in the discovery phase. In the verification phase, a more significant number of samples are used to account for the variations in the proteomes of the different studied sets. At this stage, specificity and sensitivity acquire particular importance when the researchers select the few protein biomarkers used in the assay optimization and clinical evaluation phases. In the assay optimization phase, an antibody is selected for each biomarker candidate and used to develop an immunoassay to replace the MS step in protein quantification. During the evaluation/validation phase, the assay is evaluated for analytical parameters, such as accuracy and precision. If clinical validation is successful, the protein biomarker moves to the commercialization state [111].

The path to successful protein biomarker discovery through this multistage process faces several challenges. As a result, the introduction of new protein biomarkers has been slow, and has not met the clinical need for proteomic tests [112]. Some relevant challenges include the low number of samples under study and the lack of well-designed study methods and standard protocols [113]. These variables can be optimized through more careful choices of sample types and sizes. Sample selection and processing require special consideration when performing a proteomic analysis. For example, human plasma contains tens of thousands of proteins that differ in their structures and abundances [114]. It is not always possible to identify a single or multiple disease-specific proteins that could be used as markers for a particular disease. The proteins selected in the discovery phase are often classified as false positives. This is primarily due to the low frequency of selecting low-abundance proteins and limitations in their detection [111]. Even using other biofluids—such as urine, cerebrospinal fluid, cell line homogenates, or tissue lysates—has not eliminated this complexity [111]. There are also considerations more specific to the study of IBD. Intestinal mucosal biopsies are widely used in IBD studies. Protein degradation during and after extraction might lead to the under- or over-representation of specific proteins [115]. The use of protease inhibitors that minimize protein degradation can keep this variable under control. Cell heterogeneity of the mucosal specimens is another variable that could lead to an inaccurate proteome analysis [115]. Enriching samples for specific cell types and/or organelles can lower the sample’s complexity and improve the protein identification efficiency [115,116]. The statistical power of a proteomic study is another factor that requires special attention in the biomarker discovery pipeline, especially in the discovery and verification stages. Skates et al. proposed a statistical framework for increasing the probability of identifying a biomarker that can reach the clinical validation stage [117]. According to their framework, the success of a biomarker in reaching clinical validation depends on the number of candidate proteins examined at each stage, the separation in biomarker signal between cases and controls (as measured by standard deviation), and the percentage of cases in which the biomarker is expressed. The authors provided probability tables that can be used in determining the proper sample size for a given study.

Although significant progress has been achieved in the instrumentation and sample preparation of proteomic techniques, proteomics in biomarker discovery is still in its early stages. Compared to molecular biomarkers, significant work is required to prove the utility of any protein panel as a new biomarker for IBD.

### 4.2. Epigenetics in Diagnostic Biomarkers

Epigenetic signatures are tissue- and cell-type-specific. A major challenge in IBD epigenetic studies using peripheral blood or mucosal biopsies is the cell-type heterogeneity of these specimens. Additional non-disease-specific cell types can lead to complications in interpreting the data due to interference from the different individual epigenetic features. Thus, disease-specific cell types should be purified from the mixed cell or tissue samples before analysis. However, several cell types have been linked to the pathogenesis of IBD, making the selection of disease-specific cell types in IBD a challenge. Although the techniques used in epigenetic studies are well established, they also have their limitations. Most microRNA studies use real-time quantitative PCR followed by microarrays. Although these techniques can identify a wide number of miRNAs, they are not sensitive to functionally distinct microRNA variants and slight nucleotide variations between microRNAs in the same families. They also have a low dynamic range, and cannot detect miRNAs with low expression levels [118]. Next-generation sequencing (NGS) is a high-throughput and fast method that has emerged lately as a more effective technique for identifying novel microRNAs [119].

Other challenges emerge from environmental factors, such as age, diet, and smoking, which can affect the epigenome. Hence, a well-designed study seeking to identify disease-specific variations selectively would require a careful selection of patients and controls.

## 5. Conclusions

The role of endoscopy and inflammatory biomarkers in the diagnosis of IBD has been extensively studied over the years, improving our understanding of the utility and limitations of each diagnostic tool in clinical settings. Although the combination of endoscopy and molecular tests has become a well-established diagnostic tool for IBD, there is continuing effort to find an ideal diagnostic tool that can overcome the challenges limiting the current tools. Lately, there has been growing interest in switching from using a single biomarker to the biomarker panel approach, in an effort to identify biomarkers that, together, are specific to IBD and can enable differential diagnosis of UC versus CD. This shift in research focus is evident from the increasing number of studies looking into the use of proteomics and genomics for identifying biomarker signatures. As the causes of IBD are still undetermined, with immunological, genetic, and environmental triggers having been found to contribute to disease progression [120,121,122,123], researchers also continue to search for new molecular biomarkers that are associated with these factors—especially in the context of new fecal biomarkers and serological antibodies.

## Figures and Tables

**Table 1:** Correlation of biomarkers with disease activity, determined by endoscopy.

Patient Population	Assessment of Endoscopic Disease Activity	Lactoferrin (Correlation Coefficient)	Calprotectin (Correlation Coefficient)	CRP (Correlation Coefficient)
CD	CDEIS *	0.77	0.73	0.55
CD	SES-CD **	0.19	0.48	
UC	Mayo score	0.35	0.51	
UC	Matt’s index		0.81	
CD	SES-CD	0.63	0.64	0.52
IBD			0.52	
UC	Mayo score		0.57	
CD	SES-CD	0.76	0.72	0.46
CD	CDEIS	0.87	0.83	0.61
UC	Rachmilewitz index		0.83	0.50
CD	CDEIS		0.75	0.53

* CDEIS, Crohn’s Disease Endoscopic Index of Severity; ** SES-CD, Simple Endoscopic Score for Crohn’s Disease. Adapted from James D. Lewis’s review [23]; *Gastroenterology*, Volume 140 Issue 6 Pages 1817–1826.e2; https://doi.org/10.1053/j.gastro.2010.11.058.

**Table 2:** A summary of microRNAs that are correlated with ulcerative colitis (UC#1–12) or Crohn’s disease (CD, #13–22).

#	MiRNAs	Disease Subtype	Sample Type	Techniques Used	Outcome
1	miR-19a	UC, HC	Biopsy, murine tissue	RT-qPCR	Reduced expression of miR-19a in human colon tissue with UC and DSS-treated murine colitis.
2	miR-21	UC, HC	Biopsy	RT-qPCR, ISH	Overexpression of miR-21 in UC.
3	miR-21-5p	UC, HC	Sera, rat tissue	RT-qPCR, Transfection	MiR-21-5p was downregulated in the sera and colon tissue of UC compared with healthy people and the control group.
4	miR-124	UC, HC	Biopsy	RT-qPCR	MiR-124 regulated the expression of STAT3. Reduced levels of miR-124 in colon tissues of children with active UC appeared to increase the expression and activity of STAT3.
5	miR-141	UC, HC	Biopsy	Microarray, RT-qPCR	MiR-141 played a role in the bowel inflammation of individuals with active UC via downregulation of CXCL5 expression.
6	miR-150	UC, HC	Murine model	RT-qPCR	MiR-150 was elevated and c-Myb was downregulated in the human colon with active UC compared to HC.
7	miR-155	Colitis	Murine tissue, cell culture	RT-qPCR, transfection	MiR-155 promoted the pathogenesis of experimental colitis by repressing SHIP-1 expression.
8	miR-193a-3p	UC, HC	Cell culture, biopsy	RT-qPCR, ISH	MiR-193a-3p reduced intestinal inflammation in response to microbiota.
9	miR-206	UC, HC	Cell culture, biopsy	RT-qPCR,	MiR-206 as a biomarker for response to mesalamine treatment in UC.
10	miR-21, miR-155	UC, HC	Biopsy	RT-qPCR	MiR-21 and miR-155 were highly expressed in UC.
11	miR-143, miR-145	UC, HC	Biopsy	RT-qPCR, ISH	MiR-143 and miR-145 were downregulated in UC.
12	miR-125b, miR-155, miR-223 and miR-138	UC	Biopsy	RT-qPCR, microarray	Differential expression of miR-223, miR-125b, miR-138, and miR-155 in the inflamed mucosa compared to non-inflamed mucosa and controls.
13	miR-7	CD, HC	Cell culture, biopsy	Transfection, RT-qPCR	MiR-7 modulated CD98 expression during intestinal epithelial cell differentiation.
14	miR-19b	CD, HC	Biopsy, cell culture	RT-qPCR, ISH	MiR-19b suppressed the inflammation and prevented the pathogenesis of CD.
15	miR-29b	CD	Fibroblasts	RT-qPCR	MCL-1 was modulated in CD fibrosis by miR-29b via IL-6 and IL-8
16	miR-122	CD, HC	Cell culture, biopsy	RT-qPCR, Transfection	MiR-122 reduced the expression of pro-inflammatory cytokines (TNF and IFN-γ) and promoted the release of anti-inflammatory cytokines (e.g., IL-4 and IL-10). Significant increase in miR-122 expression in cells treated with 5′-AZA.
17	miR-141	CD	Murine models, biopsy	Microarray, RT-qPCR	MiR-141 regulated colonic leukocytic trafficking by targeting CXCL12β during murine colitis and human CD.
18	miR-155	CD, HC	PBMC	RT-qPCR, transfection	MiR-155 regulated IL-10-producing CD24 CD27+ B Cells.
19	miR-200b	CD, HC	Biopsy, serum. cell culture	RT-qPCR	MiR-200b was involved in intestinal fibrosis of CD.
20	miR-590-5p	CD, HC	Human and murine tissues	RT-qPCR	Decreased miR-590-5p levels in CD.
21	miR-146a, miR-155	CD	Biopsy	RT-qPCR	MiR-146a and -155 showed increased duodenal expression in pediatric CD.
22	miR-223-3p, miR-31-5p	CD, HC	Biopsy	Nanostring	Mir-223-3p expression showed age- and sex-related effects and miR-31-5p expression was driven by location

HC: healthy controls, RT-qPCR: quantitative real-time polymerase chain reaction, Biopsy: colon tissue biopsy, ISH: in situ hybridization, PBMCs: peripheral blood mononuclear cells, DSS: dextran sodium sulfate, TNF: Tumor necrosis factor alpha. Adapted and modified from Jaslin P. James et al.’s review [92]; *Int. J. Mol. Sci*. 2020, 21, 7893; doi:10.3390/ijms21217893.

## Data Availability

No new data were generated or analyzed in support of this research.
